# Preclinical Models of Malignant Mesothelioma

**DOI:** 10.3389/fonc.2020.00101

**Published:** 2020-02-11

**Authors:** Joseph R. Testa, Anton Berns

**Affiliations:** ^1^Cancer Biology Program, Fox Chase Cancer Center, Philadelphia, PA, United States; ^2^Division of Molecular Genetics, The Netherlands Cancer Institute, Amsterdam, Netherlands

**Keywords:** malignant mesothelioma, preclinical rodent models, *in vivo* asbestos carcinogenesis, genetic driver lesions, mesothelioma inflammatory phenotype, conditional tumor suppressor gene knockout/oncogene mouse models, patient-derived xenograft models of mesothelioma

## Abstract

Rodent models of malignant mesothelioma help facilitate the understanding of the biology of this highly lethal cancer and to develop and test new interventions. Introducing the same genetic lesions as found in human mesothelioma in mice results in tumors that show close resemblance with the human disease counterpart. This includes the extensive inflammatory responses that characterize human malignant mesothelioma. The relatively fast development of mesothelioma in mice when the appropriate combination of lesions is introduced, with or without exposure to asbestos, make the autochthonous models particularly useful for testing new treatment strategies in an immunocompetent setting, whereas Patient-Derived Xenograft models are particularly useful to assess effects of inter- and intra-tumor heterogeneity and human-specific features of mesothelioma. It is to be expected that new insights obtained by studying these experimental systems will lead to new more effective treatments for this devastating disease.

## Introduction

Malignant mesothelioma (MM) is a treatment-resistant malignancy causally linked to asbestos exposure. Despite recent advances in therapeutic modalities, MM patients usually die within 1 year following diagnosis. MM is particularly lethal in patients with pleural disease, particularly those whose tumors have sarcomatoid features (1). Consequently, *in vivo* models of MM are needed to investigate MM disease pathogenesis and to provide accurate preclinical models for identifying new therapies that might move forward in clinical trials.

We here summarize where we stand with regard to existing models of MM and how they might be further improved. All the desirable features will be unlikely found in a single model, but the disease evolving in the model should mimic at least several of the salient features of human MM, such as its pathology, its gene expression patterns, the genetic driver lesions, and the inflammatory phenotype that is characteristic for MM. In view of the inflammatory phenotype of MM and the prominent role the immune system fulfills in either promoting or impairing tumor development, models exhibiting this specific feature should also be part of the armamentarium. Preferentially, the model should also exhibit a reproducible and short latency period as to permit intervention studies. The models—mostly encompassing small rodents—range from graft models in which human MM cell lines or patient-derived tumor fragments are implanted to complex conditional tumor suppressor gene knockout/oncogene mouse models.

## Somatic Genetic and Signaling Alterations in Human Mesothelioma

There is abundant evidence that inactivating somatic mutations and deletions of the tumor suppressor genes (TSGs) *BAP1, CDKN2A*, and *NF2* represent the most frequent genetic lesions in human malignant pleural mesothelioma (MPM) (2–13). Moreover, losses of these three TSGs are frequently seen in various combinations in a given MPM (7, 14). The notion that loss of these particular TSGs is so predominant implies that MPM development critically depends on the cellular signaling pathways that are guarded by these genes.

*CDKN2A* encodes p16INK4A and p14ARF, two tumor suppressors that, respectively, regulate the Rb and p53 cell cycle pathways. p14ARF is a component of the p53 pathway, and *TP53* alterations have also been observed in some MPMs (6, 15). In fact, a recent report that compared next-generation sequencing of two series of MPMs—one from The Cancer Genome Atlas (TCGA) (13) and the second from a Harvard series (12)—revealed only four “significantly mutated genes at a false discovery rate of <0.05” common to the two studies: *BAP1, NF2, TP53*, and *SETD2*, each of which showed prominent levels of inactivating nonsense, frameshift, and splice-site mutations, consistent with their putative roles as driver loss-of-function lesions in this malignancy (13). In the TCGA data set, focal deletions were found to affect several TSGs, especially *CDKN2A*, with deep, apparently homozygous deletions occurring in 36/73 (49%) tumors and single-copy losses in 5 others (7%) (13). In the Harvard series, Bueno et al. found copy number losses of *CDKN2A* in 48/95 (51%) MPMs (12). In a deletion mapping analysis, homozygous *CDKN2A* deletions were identified in 36 of 40 (90%) human MPM cell lines tested, while homozygous deletions of the adjacent locus *CDKN2B* occurred in most—i.e., 32/36—of these same cell lines (6). Experiments in mice have shown that the *Cdkn2b* also exhibits a tumor suppressor role in MPM, as its deletion concomitant with *Cdkn2a* further accelerates MPM development (our unpublished results) offering a rationale for the predominant deletion of all three tumor suppressors in this locus in MPM.

Unlike these specific TSGs, mutations of protooncogenes are seldom identified in MPM. Moreover, in the TCGA cohort, no activating mutations were observed in genes encoding components of the MAPK or PI3K/AKT pathways (13). However, both PI3K/AKT/mTOR and RAS/MAPK pathways were upregulated in this series, and they were each associated with a poor-prognosis. Moreover, despite a rarity of mutations of *PTEN* in MPM, earlier immunohistochemical (IHC) studies revealed diminished PTEN protein expression in 16 to 62% of MMs in several studies (16–18). Additionally, various receptor tyrosine kinases (RTKs) were shown to be frequently overexpressed and/or activated in MPM, resulting in activation of proliferation and pro-survival signals through the PI3K/AKT/mTOR signaling pathway (7, 19–21). Thus, it is not surprising that phospho-AKT immunostaining is observed in a high percentage (65–84%) of human MPMs (6, 16, 22, 23).

In view of the prominence of TSG inactivation and the relatively rare oncogenic gain-of-function mutations in MM, high-throughput chemical inhibitor screens and gene expression analyses have been performed in MM cell lines to identify unique vulnerabilities. Chemical screens pointed to increased sensitivity to FGFR inhibitors in a subset of the MPM cell lines. This corresponded with higher FGFR3 expression specifically in cell lines not expressing BAP1 (24). BAP1-deficient MM also showed augmented sensitivity to TRAIL (25). Furthermore, loss of BAP1 function was found associated with increased expression of EZH2, with concomitant widespread epigenetic gene silencing sensitizing the cells to EZH2 inhibitors (26), whereas the impaired argininosuccinate synthase 1 (ASS1) expression likely as a result of enhanced EZH2 levels sensitized cells to arginine deprivation (27, 28). In addition, BAP1-depleted cells showed increased sensitivity to PARP inhibition (29). Another vulnerability relates to the co-deletion of *CDKN2A* and the nearby methylthioadenosine phosphorylase (*MTAP*) gene (30), the latter rendering cells dependent on protein arginine methyltransferase (PRMT5) (31, 32). NF2 depletion leads to dysregulation of the Hippo pathway by activating the transcriptional co-activator YAP1 and its association with the TEAD family of transcription factors, resulting in up-regulation of genes that promote cell proliferation and inhibit cell death. Inhibitors that disrupt the YAP/TAZ-TEAD complex are not yet available but could serve as promising drugs in view of the strong dependence of MM on activation of the Hippo pathway (33). MM also shows overexpression of RTKs such as MET and downstream PI3K, making inhibitors targeting components of this pathway other promising therapies for this disease (21). Therefore, there are a number of potential vulnerabilities that are worth exploring both as single agents and as combinations in the various preclinical models of MM.

## Rodents as Models of Asbestos Carcinogenicity and Mesothelioma Pathogenesis

Numerous investigators have induced MM in rats and mice via injection or inhalation of asbestos fibers (34) or in hamsters through exposure to SV40 (35). Notably, several studies have shown that the MMs induced in rats via asbestos inhalation do not exhibit cytogenetic or gene expression patterns similar to those seen in their human tumor counterparts nor do they show inactivation of genes implicated as drivers in human MM (36–39). Studies in the laboratory rat, beginning in the 1960's, documented that various forms of asbestos and other mineral fibers inoculated intrapleurally/intrathoracically (IT) developed MPM (40). Erionite, the zeolite mineral fiber that is linked to the MM epidemic in near Cappadocia, Turkey (41, 42) was shown to be more carcinogenic than asbestos in IT injection or inhalation studies (43).

While the rat was favored over the mouse as a model for mineral fiber studies due to its larger pleural space for inoculation and “… its more suitable nasal passage architecture for inhalation studies, some of the early investigations did use mice for IT inoculation of amphiboles and serpentine mineral fibers,” however, fibrosis and granulomas were mainly observed (44), with occasional papillary carcinomas seen in inhalation experiments (45). Subsequent carcinogenicity studies using intraperitoneally (i.p.)-inoculated asbestos or zeolite fibers resulted in MMs in more than 20% of wild type mice (46). Over the last two decades, various laboratories have reported variable MM incidences and survival rates in wild type mice that have been injected i.p. with asbestos fibers (6, 38, 47–56), due at least in part to the use of differing types, dimensions and amounts of fibers used, whether the injections were given chronically or as a bolus injection, the length of time the animals were followed, and variations in the genetic background of the mice.

Genetically engineered mouse (GEM) models, typically harboring heterozygous whole-body germline mutations, have been used to assess whether loss of TSGs implicated in human MPM accelerate tumor formation. Different groups have performed such experiments with GEM models carrying mutations of MM-related genes. An early investigation used *Tp53-* deficient mice (38, 47), with mice injected i.p. with crocidolite weekly for 22 weeks. *Tp53*^+/−^ mice developed a high incidence (76%) of MMs (median latency, 44 weeks) vs. a32% of wild type mice (median latency, 67 weeks). Only 1/8 (12.5%) *Tp53*^−/−^ mice had a MM, with others succumbing quickly due to thymic lymphomas or hemangiosarcomas, previously reported to arise spontaneously in *Tp53*^−/−^ mice (57).

Two research groups tested whether heterozygous *Nf2* mice have increased susceptibility to the carcinogenic effects of asbestos (6, 48). Both groups independently demonstrated that *Nf2*^+/−^ mice injected i.p. with asbestos develop a high incidence and rapid onset of MMs compared wild type littermates. Notably, the normal *Nf2* allele was deleted in most MMs from the *Nf2*^+/−^ mice, consistent with biallelic inactivation, which similarly occurs in many human MPMs (6). Moreover, most MM cell lines from the *Nf2-*deficient mice showed homologous deletions of *Cdkn2a*/*Cdkn2b* and activation of Akt, recapitulating events that often occur in human MPM. Collectively, these findings are consistent with *Nf2* being a TSG that, when inactivated, acts as a primary driver in the formation of MM.

As noted previously, *CDKN2A* encodes p16INK4A and p14ARF (19Arf in the mouse). To test the relative contributions of these genes to MM formation, one study used mice with heterozygous deletions of *Cdkn2a* exon 1α (resulting in loss of p16Ink4a) or exon 1β (p19Arf), or with a deletion of exon 2 (deleting both p16Ink4a and p19Arf) (51). Both *p16Ink4a*^+/−^ mice and *p19Arf* ^+/−^ mice injected i.p. with asbestos exhibited higher incidence and more rapid onset of MM than wild type control mice. Mice heterozygous for *Cdkn2a* exon 2 showed a more accelerated rate of asbestos-induced MMs vs. mice deficient for either *p16Ink4a* or *p19Arf* separately. Together, these data indicate that each of the *Cdkn2a* gene products suppresses asbestos-induced MM, and that the combined inactivation of both gene products results in further cooperation to accelerate asbestos-induced MM development and progression.

Early Sanger sequencing studies had revealed point mutations in *BAP1* in 20–25% of sporadic human MMs (7, 8), but subsequent studies of sporadic MMs using various combinations of assays, such as quantitative real-time PCR, targeted comparative genomic hybridization, next generation sequencing, and/or multiplex ligation-dependent probe amplification platforms demonstrated *BAP1* alterations in up to 60–65% of MMs (9–11). Most of the alterations not detected by Sanger sequencing were large deletions.

In addition to somatic changes, it is now well-established that *BAP1* mutation carriers are predisposed to MM and a variety of other tumors (8, 58). The use of *Bap1* knockout models has shown that heterozygosity in the germline predisposes to asbestos-induced MM (53, 59), and similar results were obtained with two knock-in models (54) that harbored different germline mutations that were identical to the ones found in two BAP1 tumor predisposition syndrome (BAP1-TPDS) families that exhibited a very high incidence of MM (8). MM cells from *Bap1*^+/−^ mice showed biallelic inactivation of *Bap1* (53). Collectively, these data indicate that human *BAP1* mutation carriers have are more prone to the carcinogenic effects of asbestos, even when exposed to small amounts of these fibers (59), when compared to the general population.

Other work has recently demonstrated cooperation between *Nf2* and *Cdkn2a* in MM development in asbestos-exposed *Nf2*^+/−^;*Cdkn2a*^+/−^ mice, which exhibited significantly hastened tumor onset and disease progression vs. similarly exposed *Nf2*^+/−^ and wild-type cohorts (56). These studies also showed that tumors from *Nf2*^+/−^;*Cdkn2a*^+/−^ mice had enhanced metastatic potential and an increased cancer stem cell population, in connection with p53/miR-34a-dependent activation of c-Met.

Since chronic inflammation may contribute to the formation of many types of malignancy, including MM, some investigators have employed mouse models for studies of asbestos-mediated inflammation. In one such study, *Nf2*^+/−^;*Cdkn2a*^+/−^ mice were used to test if inflammation-related IL-1β release promotes MM formation (55). Exposure of *Nf2*^+/−^;*Cdkn2a*^+/−^ mice to asbestos in the presence of an IL-1 receptor (IL-1R) antagonist known as anakinra resulted in a significant delay MM development compared to that of asbestos-exposed mice given a vehicle control, i.e., 33 vs. ~22.5 weeks, respectively (55). Overall, this work suggested that inflammation-related IL-1β/IL-1R signaling is linked to the formation of asbestos-induced MM. Moreover, the data demonstrate the usefulness of this model for gene-environment and/or “chemoprevention” studies.

Another mouse model, MexTAg, has been used to demonstrate co-carcinogenicity between asbestos and SV40. The investigators used the mesothelin gene promoter to express SV40 large T antigen specifically in the mesothelial lining (49, 60). Several MexTAg mouse lines were created with varying copies (1–100) of the oncogenic transgene. The animals generally do not develop spontaneous MM. However, after i.p. injection of asbestos, 100% of the MexTAg mice developed MM, with disease onset occurring after 20–40 weeks vs. after 50–100 weeks in the ~25% of wild type mice that developed MM. The investigators concluded that MexTAg mice are well-suited not only basic research, but also for testing the potential of dietary or pharmacological chemoprevention studies of MM (49). To illustrate the utility of MexTAg mice for preclinical studies, Robinson et al. tested the effects of gemcitabine, a cytotoxic drug that has been shown to have some efficacy in the human disease (60). MexTAg mice treated with vehicle had a median survival of 33 vs. 48 weeks in the gemcitabine-treated cohort. In another investigation with MexTAg mice, treatment with celecoxib, a COX-2 inhibitor, did not diminish the rate of asbestos-induced MM, despite the fact that COX-2 is frequently overexpressed in human MM and correlates with poor prognosis (60). While the MexTAg model has several advantages (100% MM penetrance, short median survival), it does not have any of the genetic hallmarks attributed to the human disease, and a causative association between SV40 and human MM is now disproven (61, 62). However, in one study, gene expression profiling of MMs from MexTAg mice “…had a concordant set of deregulated genes compared to normal mesothelial cells that overlapped with the deregulated genes between human MMs and mesothelial cells” (63).

## Conditional Mouse Models of Mesothelioma as Preclinical Tools

Since specific genetic driver lesions had been repeatedly found to be associated with human MM by the year 2008, particularly alterations of the *CDKN2A, NF2*, and *TP53*, Jongsma et al. decided to establish whether various genetic alterations affecting the same signaling pathways that are dysregulated in the human disease counterpart might similarly induce MM in rodents in the absence of carcinogenic exposure to asbestos (64). Thus, they generated a variety of mutant mice carrying deletions in the Nf2/merlin, p53, and/or Ink4a pathways, hypothesizing that mice with one or more of these combinations might represent an appropriate model of human MM. To avoid possible issues such as embryonic lethality due to germline homozygous deletion of one or more targeted genes, mice with conditional knockout (CKO) of various TSGs were used in combination with the Cre-LoxP system (65). Locotemporal inactivation of the TSG(s) was carried out by injecting adenoviruses expressing the Cre recombinase (65). Upon injecting adeno-Cre into the pleural space of Rosa26 LacZ reporter mice, the investigators demonstrated expression of β-galactosidase specifically in the mesothelium (64). Moreover, MPMs arose in both *Nf2;Tp53* and *Nf2;p16Ink4a/p19Arf* CKO mice at a high frequency and short latency (20 and 30 weeks, respectively) following IT inoculation of adeno-Cre, and the tumors closely mimicked the phenotype of human MPM. Thus, these mice hold promise as a rapid, non-carcinogenic model system for preclinical selection of new combination therapies and for testing novel targeted agents.

BAP1-TPDS patients with MM have a significantly better long-term survival compared to sporadic MM patients, i.e., those without a heritable variant (11, 66). However, it remained unclear whether somatic mutations/deletions of *BAP1* have a similarly favorable prognosis in sporadic MM, or if somatic *BAP1* alterations are a poor prognostic marker, as is the case for uveal melanoma and clear cell renal cell carcinoma (67, 68). Furthermore, although most human MMs exhibit somatic alterations of *BAP1, NF2*, and/or *CDKN2A*—with 25/74 cases of MPM in the TCGA series having alterations of all three TSGs in combination (13)—it was not known if loss of *BAP1* could cooperate with the inactivation of *NF2* and/or *CDKN2A* to initiate a more aggressive form of MM. To address this possibility experimentally, Kukuyan et al. used CKO models, including a *Bap1*^*f*/*f*^ mouse they generated (69). Various combinations of deletions of *Bap1, Cdkn2a*, and *Nf2* were introduced in the pleural cavity of the mice, focusing on the contribution of Bap1 loss. While homozygous CKO of any one of these TSGs alone gave rise to few or no MMs—similar to the results of Jongsma et al. (64)—deletion of *Bap1* cooperated with deletion of either *Nf2* or *Cdkn2a* to promote MM formation in about 20% of double-CKO mice. In contrast, a much higher incidence (22/26, 85%) of MMs was observed in *Bap1*^*f*/*f*^*;Nf2*^*f*/*f*^*;Cdkn2a*^*f*/*f*^ mice injected IT with adeno-Cre (triple-CKO mice). Onset of MM was rapid in the triple-CKO mice (median survival, 12 weeks), and tumors from these mice were consistently high-grade and invasive. With regard to histological subtype, notably no epithelioid MMs were observed with any of the mouse genotypes. Sarcomatoid MMs predominated, with the only exception being the *Bap1;Nf2* double-CKO cohort, in which 6 of 7 MMs showed mixed (biphasic) histology. The MMs observed in triple-CKO mice showed enrichment for genes that are transcriptionally controlled by the polycomb repressive complex 2 (PRC2) (69). The findings suggested that loss of *Bap1* contributes to MM progression, at least partially, via loss of PRC2-mediated repression of oncogenic target genes that were identified, suggesting a novel avenue for therapeutic intervention (69).

To explore the role of individual components of the *Cdkn2a* locus by comparing models in which *Cdkn2a* (including *p19Arf*) were disrupted with or without concomitant loss of *Cdkn2b* Badhai et al. showed that the additional disruption of *Cdkn2b* further added to the aggressiveness of the resulting MMs, providing also an explanation for the predominance of deletion of the complete *CDKN2A-CDKN2B* locus in human MM over point mutations in *CDKN2A* (Badhai et al., submitted).

Because *CDKN2A* deletions encompassing the sequence encoding p14ARF, a component of the p53 pathway, have been documented in 90% of human MM cell lines (6) and *TP53* is altered in about 15% of primary MMs, and because the PI3K/PTEN/AKT pathway is activated in most human MPMs, Sementino et al. decided to determine if alterations affecting the same pathways would also induce MM in mice (70). This was thought worthwhile, given that p53 helps mediate the DNA damage response and that AKT regulates neoplastic cell survival and therapeutic resistance. The investigators demonstrated that while neither adeno-Cre-mediated homozygous deletion of *Tp53* or *Pten* alone in the mesothelium was sufficient to induce MM formation, compound deletion of these two TSGs resulted in rapid, aggressive peritoneal and pleural MMs (median latency: 9 and 19 weeks, respectively). A longer term follow-up study of the *Tp53*^*f*/*f*^ cohort revealed MMs in 0/12 mice injected with adeno-Cre i.p. and 0/10 mice injected IT; among the *Pten*^*f*/*f*^ cohort, MMs were observed in 0/12 mice injected i.p. and 1/10 injected IT (Sementino et al., unpublished data). In the *Pten*^*f*/*f*^*;Tp53*^*f*/*f*^ cohort, 23/25 (92%) mice injected i.p. developed MM, whereas 19/34 (56%) mice injected IT showed MM, with 14 histiocytic sarcomas also seen in this group.

Given the high penetrance and rapid development of MMs in *Pten*^*f*/*f*^*;Tp53*^*f*/*f*^ mice inoculated i.p., and the frequent involvement of p14ARF/p53 and PI3K/PTEN/AKT pathways in human MM, this GEM model holds promise for preclinical work. However, this model does have certain limitations, such as for testing agents designed to reactivate the normal cellular functions of Pten and Tp53. For instance, given that this model has homozygous loss of *Tp53*, this precludes studies of a drug such as RITA, which reactivates p53's pro-apoptotic function in tumor cells that preserve expression of mutant or wild-type p53 (71). To elude this issue using an agent such as RITA, this mouse model might be modified such that only a single *Tp53* allele were deleted, i.e., by using *Pten*^*f*/*f*^*;Tp53*^+/*f*^ mice. A second shortcoming with regard to the translational relevance of the *Pten*^*f*/*f*^*;Tp53*^*f*/*f*^ model is that somatic mutations of other TSGs considered to be hallmarks in human MM progression usually do not occur in tumors from these animals. However, the fact that the MMs in this model repeatedly show sarcomatoid or biphasic histology with very short latency, especially in mice injected i.p., provides advantages for certain preclinical applications.

## Graft Models of Mesothelioma

Many human MM cell lines have been established over the years and used in numerous *in vitro* studies. They are also exploited for *in vivo* experiments, usually for testing their tumorigenicity and the efficacy of small molecule inhibitors as a prelude for evaluating these compounds in clinical trials. Due to often long-term *in vitro* propagation, these cell lines have invariably acquired (epi)genetic alterations that facilitate their propagation in cell culture, resulting in new vulnerabilities and resistance features. This is one of the reasons why treatments that are effective in these graft models often do not well-translate to human. Furthermore, the requirement to use immunodeficient mice as a host for these graft experiments complicates assessment of immunomodulating effects. Patient-derived xenograft (PDX) models, in which tumor fragments are grafted directly into immunodeficient recipient hosts, more closely resemble the human condition and usually retain their human stromal components for a number of passages. The capacity to establish PDX lines also correlates with the aggressiveness of the tumor in man (72). Studies in PDX models permit addressing specific questions that are difficult to assess in solely mouse based models such as inter- and intra-tumor heterogeneity as well as features imposed by the distinct genetic backgrounds (73). As potential drawbacks, we note that propagation has to be performed in immunodeficient backgrounds and retrofitting these models with a functional human immune system from the patient from which the tumor was obtained (humanized models) is still in an early stage of development (74) and also practically very demanding.

Experienced investigators seeking a mouse model that may faithfully reflect human MPM pathobiology may also find an orthotopic, intrapleural model such as the one described by Servais et al. (75) useful for preclinical therapeutic studies. This tumor model recapitulates human pleural anatomy/microenvironment and can be used in combination with quantitative, non-invasive imaging for bioluminescent monitoring of tumor burden. The parietal pleural surface contains lymphatics that offer escape of MM cells into the systemic circulation, and this immunocompetent orthotopic model of pleural cancer permit studies of inflammation on tumor progression as well (75). However, as noted by the authors, for studies of therapies targeting human antigens, immunodeficient models are required in order to perform studies on xenografted human cancer cell lines.

## Are we Missing Anything?

First of all, it is worth emphasizing that the choice of the model depends on the question asked. Furthermore, a mouse is not a “small human” and we need to accept that we cannot simply extrapolate findings from such a model to the human condition. However, models can teach us important biological principles and can provide us with therapeutic concepts worth testing in clinical settings notwithstanding the evolutionary distance between man and mouse. Where possible, we should try to align the model on the basis of molecular aberrations found in humans, e.g., by introducing similar driver lesions in the right target cell and using comparable external carcinogens if applicable, e.g., asbestos. Evidently, PDX models might be very valuable to assess intrinsic tumor heterogeneity and to evaluate their response to drug combinations. For immunotherapy studies, it will be important to use a model with a functional immune system. To permit effective immunotherapy studies in MM mouse models, it will be important to establish these in a defined genetic background (e.g., BL6, the “work horse” of immunologists) in order to permit isogenic graft studies. Fortunately, current Crispr/Cas9 engineering has made the generation of complex conditional MM models relatively easy. This should facilitate the testing of new promising intervention strategies for this highly lethal cancer.

## Author Contributions

JT and AB wrote the paper and are accountable for the content of the work.

### Conflict of Interest

JT has a patent on *BAP1* mutation testing and has provided legal consultation regarding the role of germline mutations of *BAP1* in mesothelioma. The remaining author declares that the research was conducted in the absence of any commercial or financial relationships that could be construed as a potential conflict of interest.

## References

[1] Amin W Linkov F Landsittel DP Silverstein JC Bashara W Gaudioso C. Factors influencing malignant mesothelioma survival: a retrospective review of the National Mesothelioma Virtual Bank cohort [version 3]. F1000Res. (2018) 7:1184. 10.12688/f1000research.15512.2 30410729 PMC6198263

[2] Cheng JQ Jhanwar SC Klein WM Bell DW Lee W-C Altomare DA. *p16* alterations and deletion mapping of 9p21-p22 in malignant mesothelioma. Cancer Res. (1994) 54:5547–5551. 7923195

[3] Xio S Li D Vijg J Sugarbaker DJ Corson JM Fletcher JA. Codeletion of p15 and p16 in primary malignant mesothelioma. Oncogene. (1995) 11:511–5. 7630635

[4] Bianchi AB Mitsunaga S-I Cheng JQ Klein WM Jhanwar SC Seizinger B. High frequency of inactivating mutations in the neurofibromatosis type 2 gene (*NF2*) in primary malignant mesotheliomas. Proc Natl Acad Sci USA. (1995) 92:10854–8. 10.1073/pnas.92.24.10854 7479897 PMC40529

[5] Sekido Y Pass HI Bader S Mew DJY Christman MF Gazdar AF Neurofibromatosis type 2 (*NF2*) gene is somatically mutated in mesothelioma but not in lung cancer. Cancer Res. (1995) 55:1227–31. 7882313

[6] Altomare DA Vaslet CA Skele KL De Rienzo A Devarajan K Jhanwar SC. A mouse model recapitulating molecular features of human mesothelioma. Cancer Res. (2005) 65:8090–5. 10.1158/0008-5472.CAN-05-2312 16166281

[7] Bott M Brevet M Taylor BS Shimizu S Ito T Wang L. The nuclear deubiquitinase BAP1 is commonly inactivated by somatic mutations and 3p21.1 losses in malignant pleural mesothelioma. Nat Genet. (2011) 43:668–72. 10.1038/ng.855 21642991 PMC4643098

[8] Testa JR Cheung M Pei J Below JE Tan Y Sementino E. Germline *BAP1* mutations predispose to malignant mesothelioma. Nat Genet. (2011) 43:1022–5. 10.1038/ng.912 21874000 PMC3184199

[9] Yoshikawa Y Sato A Tsujimura T Emi M Morinaga T Fukuoka K. Frequent inactivation of the *BAP1* gene in epithelioid-type malignant mesothelioma. Cancer Sci. (2012) 103:868–74. 10.1111/j.1349-7006.2012.02223.x 22321046 PMC7659203

[10] Lo Iacono M Monica V Righi L Grosso F Libener R Vatrano S. Targeted next-generation sequencing of cancer genes in advanced stage malignant pleural mesothelioma: a retrospective study. J Thorac Oncol. (2015) 10:492–9. 10.1097/JTO.0000000000000436 25514803

[11] Nasu M Emi M Pastorino S Tanji M Powers A Luk H. High incidence of somatic *BAP1* alterations in sporadic malignant mesothelioma. J Thorac Oncol. (2015) 10:565–76. 10.1097/JTO.0000000000000471 25658628 PMC4408084

[12] Bueno R Stawiski EW Goldstein LD Durinck S De Rienzo A Modrusan Z. Comprehensive genomic analysis of malignant pleural mesothelioma identifies recurrent mutations, gene fusions and splicing alterations. Nat Genet. (2016) 48:407–16. 10.1038/ng.3520 26928227

[13] Hmeljak J Sanchez-Vega F Hoadley KA Shih J Stewart C Heiman D. Integrative molecular characterization of malignant pleural mesothelioma. Cancer Discov. (2018) 8:1548–65. 10.1158/2159-8290.CD-18-0804 30322867 PMC6310008

[14] Cheung M Testa JR. *BAP1*, a tumor suppressor gene driving malignant mesothelioma. Transl Lung Cancer Res. (2017) 6:270–8. 10.21037/tlcr.2017.05.03 28713672 PMC5504107

[15] Cote RJ Jhanwar SC Novick S Pellicer A Genetic alterations of the p53 gene are a feature of malignant mesothelioma. Cancer Res. (1991) 51:5410–6. 1913660

[16] Garland LL Rankin C Gandara DR Rivkin SE Scott KM Nagle RB. Phase II study of erlotinib in patients with malignant pleural mesothelioma: a Southwest Oncology Group Study. J Clin Oncol. (2007) 25:2406–13. 10.1200/JCO.2006.09.7634 17557954

[17] Opitz I Soltermann A Abaecherli M Hinterberger M Probst-Hensch N Stahel R. PTEN expression is a strong predictor of survival in mesothelioma patients. Eur J Cardiothorac Surg. (2008) 33:502–6. 10.1016/j.ejcts.2007.09.045 18248818

[18] Agarwal V Campbell A Beaumont KL Cawkwell L Lind MJ. PTEN protein expression in malignant pleural mesothelioma. Tumour Biol. (2013) 34:847–51. 10.1007/s13277-012-0615-9 23242608

[19] Perrone F Jocolle G Pennati M Deraco M Baratti D Brich S. Receptor tyrosine kinase and downstream signalling analysis in diffuse malignant peritoneal mesothelioma. Eur J Cancer. (2010) 46:2837–48. 10.1016/j.ejca.2010.06.130 20692828

[20] Menges CW Chen Y Mossman BT Chernoff J Yeung AT Testa JR. A phosphotyrosine proteomic screen identifies multiple tyrosine kinase signaling pathways aberrantly activated in malignant mesothelioma. Genes Cancer. (2010) 1:493–505. 10.1177/1947601910375273 20672017 PMC2909631

[21] Kanteti R Dhanasingh I Kawada I Lennon FE Arif Q Bueno R. MET and PI3K/mTOR as a potential combinatorial therapeutic target in malignant pleural mesothelioma. PLoS ONE. (2014) 9:e105919. 10.1371/journal.pone.0105919 25221930 PMC4164360

[22] Cedres S Ponce-Aix S Pardo-Aranda N Navarro-Mendivil A Martinez-Marti A Zugazagoitia J. Analysis of expression of PTEN/PI3K pathway and programmed cell death ligand 1 (PD-L1) in malignant pleural mesothelioma (MPM). Lung Cancer. (2016) 96:1–6. 10.1016/j.lungcan.2016.03.001 27133741

[23] Altomare DA Testa JR. Perturbations of the AKT signaling pathway in human cancer. Oncogene. (2005) 24:7455–64. 10.1038/sj.onc.1209085 16288292

[24] Quispel-Janssen JM Badhai J Schunselaar L Price S Brammeld J Iorio F. Comprehensive pharmacogenomic profiling of malignant pleural mesothelioma identifies a subgroup sensitive to FGFR inhibition. Clin Cancer Res. (2018) 24:84–94. 10.1158/1078-0432.CCR-17-1172 29061644

[25] Kolluri KK Alifrangis C Kumar N Ishii Y Price S Michaut M. Loss of functional BAP1 augments sensitivity to TRAIL in cancer cells. eLife. (2018) 7:e30224. 10.7554/eLife.30224 29345617 PMC5773178

[26] LaFave LM Beguelin W Koche R Teater M Spitzer B Chramiec A. Loss of BAP1 function leads to EZH2-dependent transformation. Nat Med. (2015) 21:1344–9. 10.1038/nm.3947 26437366 PMC4636469

[27] Delage B Fennell DA Nicholson L McNeish I Lemoine NR Crook T. Arginine deprivation and argininosuccinate synthetase expression in the treatment of cancer. Int J Cancer. (2010) 126:2762–72. 10.1002/ijc.25202 20104527

[28] Yap TA Aerts JG Popat S Fennell DA. Novel insights into mesothelioma biology and implications for therapy. Nat Rev Cancer. (2017) 17:475–88. 10.1038/nrc.2017.42 28740119

[29] Parrotta R Okonska A Ronner M Weder W Stahel R Penengo L. A novel BRCA1-associated protein-1 isoform affects response of mesothelioma cells to drugs impairing BRCA1-mediated DNA repair. J Thorac Oncol. (2017) 12:1309–19. 10.1016/j.jtho.2017.03.023 28389374

[30] Illei PB Ladanyi M Rusch VW Zakowski MF. The use of *CDKN2A* deletion as a diagnostic marker for malignant mesothelioma in body cavity effusions. Cancer. (2003) 99:51–6. 10.1002/cncr.10923 12589646

[31] Mavrakis KJ McDonald ER 3rd Schlabach MR Billy E Hoffman GR deWeck A. Disordered methionine metabolism in MTAP/CDKN2A-deleted cancers leads to dependence on PRMT5. Science. (2016) 351:1208–13. 10.1126/science.aad5944 26912361

[32] Kryukov GV Wilson FH Ruth JR Paulk J Tsherniak A Marlow SE. MTAP deletion confers enhanced dependency on the PRMT5 arginine methyltransferase in cancer cells. Science. (2016) 351:1214–8. 10.1126/science.aad5214 26912360 PMC4997612

[33] Moroishi T Hansen CG Guan KL. The emerging roles of YAP and TAZ in cancer. Nat Rev Cancer. (2015) 15:73–9. 10.1038/nrc3876 25592648 PMC4562315

[34] Cheung M Menges CW Testa JR In: Testa JR, editor. Asbestos and Mesothelioma. Heidelberg: Springer Verlag (2017). p. 175–95. 10.1007/978-3-319-53560-9_8

[35] Cicala C Pompetti F Carbone M SV40 induces mesothelioma in hamsters. Am J Pathol. (1993) 142:1524–33. 8388174 PMC1886912

[36] Craighead JE Akley NJ Gould LB Libbus GL Characteristics of tumors and tumor cells cultured from experimental asbestos-induced mesotheliomas in rats. Am J Path. (1987) 24:448–62. PMC1899808 2827488

[37] Libbus BL Craighead JE. Chromosomal translocations with specific breakpoints in asbestos-induced rat mesotheliomas. Cancer Res. (1988) 48:6455–61. 3180061

[38] Marsella JM Liu BL Vaslet CA Kane AB. Susceptibility of *p53*-deficient mice to induction of mesothelioma by crocidolite asbestos fibers. Environ Health Perspect. (1997) 105:1069–72. 10.2307/3433511 9400702 PMC1470134

[39] Sandhu H Dehnen W Roller M Abel J Unfried K. mRNA expression patterns in different stages of asbestos-induced carcinogenesis in rats. Carcinogenesis. (2000) 21:1023–9. 10.1093/carcin/21.5.1023 10783328

[40] Wagner JC Berry G Mesotheliomas in rats following inoculation with asbestos. Br J Cancer. (1969) 23:567–81. 10.1038/bjc.1969.70 5360333 PMC2008422

[41] Baris YI Sahin AA Ozesmi M Kerse I Ozen E Kolacan B. An outbreak of pleural mesothelioma and chronic fibrosing pleurisy in the village of Karain/Urgup in Anatolia. Thorax. (1978) 33:181–92. 10.1136/thx.33.2.181 663877 PMC470868

[42] Carbone M Emri S Dogan AU Steele I Tuncer M Pass HI. A mesothelioma epidemic in Cappadocia: scientific developments and unexpected social outcomes. Nat Rev Cancer. (2007) 7:147–54. 10.1038/nrc2068 17251920

[43] Wagner JC Skidmore JW Hill RJ Griffiths DM. Erionite exposure and mesotheliomas in rats. Br J Cancer. (1985) 51:727–30. 10.1038/bjc.1985.108 2986668 PMC1977068

[44] Davis JM. The long term fibrogenic effects of chrysotile and crocidolite asbestos dust injected into the pleural cavity of experimental animals. Br J Exp Pathol. (1970) 51:617–27. 5493143 PMC2072361

[45] Reeves AL Puro HE Smith RG. Inhalation carcinogenesis from various forms of asbestos. Environ Res. (1974) 8:178–202. 10.1016/0013-9351(74)90050-4 4455505

[46] Suzuki Y Kohyama N. Malignant mesothelioma induced by asbestos and zeolite in the mouse peritoneal cavity. Environ Res. (1984) 35:277–92. 10.1016/0013-9351(84)90136-1 6092048

[47] Vaslet CA Messier NJ Kane AB. Accelerated progression of asbestos-induced mesotheliomas in heterozygous p53(+/–) mice. Toxicol Sci. (2002) 68:331–8. 10.1093/toxsci/68.2.331 12151629

[48] Fleury-Feith J Lecomte C Renier A Matrat M Kheuang L Abramowski V. Hemizygosity of *Nf2* is associated with increased susceptibility to asbestos-induced peritoneal tumours. Oncogene. (2003) 22:3799–805. 10.1038/sj.onc.1206593 12802287

[49] Robinson C van Bruggen I Segal A Dunham M Sherwood A Koentgen F. A novel SV40 TAg transgenic model of asbestos-induced mesothelioma: malignant transformation is dose dependent. Cancer Res. (2006) 66:10786–94. 10.1158/0008-5472.CAN-05-4668 17108115

[50] Altomare DA Menges CW Pei J Zhang L Skele-Stump KL Carbone M. Activated TNF-alpha/NF-kappaB signaling via down-regulation of Fas-associated factor 1 in asbestos-induced mesotheliomas from Arf knockout mice. Proc Natl Acad Sci USA. (2009) 106:3430–5. 10.1073/pnas.0808816106 19223589 PMC2644256

[51] Altomare DA Menges CW Xu J Pei J Zhang L Tadevosyan A. Losses of both products of the *Cdkn2a/Arf* locus contribute to asbestos-induced mesothelioma development and cooperate to accelerate tumorigenesis. PLoS ONE. (2011) 6:e18828. 10.1371/journal.pone.0018828 21526190 PMC3079727

[52] Chow MT Tschopp J Möller A Smyth MJ. NLRP3 promotes inflammation-induced skin cancer but is dispensable for asbestos-induced mesothelioma. Immunol Cell Biol. (2012) 90:983–6. 10.1038/icb.2012.46 23010873

[53] Xu J Kadariya Y Cheung M Pei J Talarchek J Sementino E. Germline mutation of *Bap1* accelerates development of asbestos-induced malignant mesothelioma. Cancer Res. (2014) 74:4388–97. 10.1158/0008-5472.CAN-14-1328 24928783 PMC4165574

[54] Kadariya Y Cheung M Xu J Pei J Sementino E Menges CW. Bap1 is a bona fide tumor suppressor: genetic evidence from mouse models carrying heterozygous germline Bap1 mutations. Cancer Res. (2016) 76:2836–44. 10.1158/0008-5472.CAN-15-3371 26896281 PMC4873414

[55] Kadariya Y Menges CW Talarchek J Cai KQ Klein-Szanto AJ Pietrofesa RA. Inflammation-related IL1β/IL1R signaling promotes the development of asbestos-induced malignant mesothelioma. Cancer Prev Res. (2016) 9:406–14. 10.1158/1940-6207.CAPR-15-0347 26935421 PMC4854753

[56] Menges CW Kadariya Y Altomare D Talarchek J Neumann-Domer E Wu Y. Tumor suppressor alterations cooperate to drive aggressive mesotheliomas with enriched cancer stem cells via a p53-miR-34a-c-Met axis. Cancer Res. (2014) 74:1261–71. 10.1158/0008-5472.CAN-13-2062 24371224 PMC3945416

[57] Donehower LA Harvey M Vogel H McArthur MJ Montgomery CA Jr Park SH. Effects of genetic background on tumorigenesis in p53-deficient mice. Mol Carcinog. (1995) 14:16–22. 10.1002/mc.2940140105 7546219

[58] Walpole S Pritchard AL Cebulla CM Pilarski R Stautberg M Davidorf FH. Comprehensive study of the clinical phenotype of germline BAP1 variant-carrying families worldwide. J Natl Cancer Inst. (2018) 110:1328–41. 10.1093/jnci/djy171 30517737 PMC6292796

[59] Napolitano A Pellegrini L Dey A Larson D Tanji M Flores EG. Minimal asbestos exposure in germline BAP1 heterozygous mice is associated with deregulated inflammatory response and increased risk of mesothelioma. Oncogene. (2016) 35:1996–2002. 10.1038/onc.2015.243 26119930 PMC5018040

[60] Robinson C Walsh A Larma I O'Halloran S Nowak AK Lake RA. MexTAg mice exposed to asbestos develop cancer that faithfully replicates key features of the pathogenesis of human mesothelioma. Eur J Cancer. (2011) 47:151–61. 10.1016/j.ejca.2010.08.015 20850297

[61] Lopez-Rios F Illei PB Rusch V Ladanyi M. Evidence against a role for SV40 infection in human mesotheliomas and high risk of false-positive PCR results owing to presence of SV40 sequences in common laboratory plasmids. Lancet. (2004) 364:1157–66. 10.1016/S0140-6736(04)17102-X 15451223

[62] Hübner R Van Marck E. Reappraisal of the strong association between simian virus 40 and human malignant mesothelioma of the pleura (Belgium). Cancer Causes Control. (2002) 13:121–9. 10.1023/A:1014321729038 11936818

[63] Robinson C Dick IM Wise MJ Holloway A Diyagama D Robinson BW. Consistent gene expression profiles in MexTAg transgenic mouse and wild type mouse asbestos-induced mesothelioma. BMC Cancer. (2015) 15:983. 10.1186/s12885-015-1953-y 26680231 PMC4683914

[64] Jongsma J van Montfort E Vooijs M Zevenhoven J Krimpenfort P van der Valk M A conditional mouse model for malignant mesothelioma. Cancer Cell. (2008) 12:261–71. 10.1016/j.ccr.2008.01.030 18328429

[65] Akagi K Sandig V Vooijs M Van der Valk M Giovannini M Strauss M. Cre-mediated somatic site-specific recombination in mice. Nucleic Acids Res. (1997) 25:1766–73. 10.1093/nar/25.9.1766 9108159 PMC146656

[66] Ohar JA Cheung M Talarchek J Howard SE Howard TD Hesdorffer M. Germline *BAP1* mutational landscape of asbestos-exposed malignant mesothelioma patients with family history of cancer. Cancer Res. (2016) 76:206–15. 10.1158/0008-5472.CAN-15-0295 26719535 PMC4715907

[67] Harbour JW Onken MD Roberson ED Duan S Cao L Worley LA. Frequent mutation of *BAP1* in metastasizing uveal melanomas. Science. (2010) 330:1410–3. 10.1126/science.1194472 21051595 PMC3087380

[68] Pena-Llopis S Vega-Rubin-de-Celis S Liao A Leng N Pavia-Jimenez A Wang S. BAP1 loss defines a new class of renal cell carcinoma. Nat Genet. (2012) 44:751–9. 10.1038/ng.2323 22683710 PMC3788680

[69] Kukuyan AM Sementino E Kadariya Y Menges CW Cheung M Tan Y. Inactivation of *Bap1* cooperates with losses of *Nf2* and *Cdkn2a* to drive the development of pleural malignant mesothelioma in conditional mouse models. Cancer Res. (2019) 79:4113–23. 10.1158/0008-5472.CAN-18-4093 31151962 PMC6697648

[70] Sementino E Menges CW Kadariya Y Peri S Xu J Liu Z. Inactivation of *Tp53* and *Pten* drives rapid development of pleural and peritoneal malignant mesotheliomas. J Cell Physiol. (2018) 233:8952–61. 10.1002/jcp.26830 29904909 PMC6168364

[71] Zhao CY Grinkevich VV Nikulenkov F Bao W Selivanova G. Rescue of the apoptotic-inducing function of mutant p53 by small molecule RITA. Cell Cycle. (2010) 9:1847–55. 10.4161/cc.9.9.11545 20436301

[72] Wu L Allo G John T Li M Tagawa T Opitz I. Patient-derived xenograft establishment from human malignant pleural mesothelioma. Clin Cancer Res. (2017) 23:1060–7. 10.1158/1078-0432.CCR-16-0844 27683181

[73] Nabavi N Wei J Lin D Collins CC Gout PW Wang Y. Pre-clinical models for malignant mesothelioma research: from chemical-induced to patient-derived cancer xenografts. Front Genet. (2018) 9:232. 10.3389/fgene.2018.00232 30022998 PMC6040159

[74] Herndler-Brandstetter D Shan L Yao Y Stecher C Plajer V Lietzenmayer M. Humanized mouse model supports development, function, and tissue residency of human natural killer cells. Proc Natl Acad Sci USA. (2017) 114:E9626–34. 10.1073/pnas.1705301114 29078283 PMC5692533

[75] Servais EL Colovos C Kachala SS Adusumilli PS Pre-clinical mouse models of primary and metastatic pleural cancers of the lung and breast and the use of bioluminescent imaging to monitor pleural tumor burden. Curr Protoc Pharmacol. (2011) 54:14.21.1–18. 10.1002/0471141755.ph1421s54 PMC4547936 21898334

